# Supplementary material for: An improved diagnostic method for taurodontism and a comparative study on its effectiveness evaluation
Source: PLoS One. 2024 May 7;19(5):e0302810. doi: 10.1371/journal.pone.0302810 (PMC11075821; doi:10.1371/journal.pone.0302810)
Supplement: Attachment — Submitted filename: PONE-D-23-41512_reviewer (1).docx [file pone.0302810.s002.docx]

**PLOS ONE**

## An improved diagnostic method for taurodontism and a comparative study on its effectiveness evaluation

--Manuscript Draft--

| **Manuscript Number:** | PONE-D-23-41512 |
| --- | --- |
| **Article Type:** | Research Article |
| **Full Title:** | An improved diagnostic method for taurodontism and a comparative study on its effectiveness evaluation |
| **Short Title:** | An improved diagnostic method for taurodontism. |
| **Corresponding Author:** | Yunmeng Da Hebei Eye Hospital Xingtai, CHINA |
| **Keywords:** | Taurodontism; Diagnostic method; CEJ; Root bifurcation; Taurodontism index; Crown-body/root ratios. |
| **Abstract:** | Objective: Since the two commonly used diagnostic methods of taurodontism were susceptible to aging changes, mastication wear and other factors, this study proposed an improved diagnostic method of taurodontism, and compared with the previous two methods, as a supplementary for the taurodontism diagnosis.  Metho ds: The patients included were age 10–89 years and admitted to the Department of Stomatology of Hebei Eye Hospital from June 1, 2022 to May 31, 2023. 80 CBCT images were selected and divided into 4 groups: 10-29 years, 30-49 years, 50-69 years, and 70-89 years, with 20 images in each group. Select the  right mandibular first molars as the measurement object. Firstly, \|BD\| and taurodontism index (TI) related parameters \|AB\| and \|AC\| were measured using Shifman and Chanannel's method and the length of crow-body(CB) and root (R)  was measured by Seow and Lai's method. Then the improved method took the length of CEJ to root bifurcation point（Body，B） and the root length（Root，R）as the measurement objects. Finally, TI ,CB/R ratios and B/R ratios was calculated according to the formulas given. One-way ANOVA analysis was mainly used to compare the differences in the values, indexes or ratios of taurodontism among different age groups (P<0.05).  Results: Among groups, with the increase of age, the values of \|BD\| and TI decreased significantly (p<0.01). For CB/R ratios, there was a downward trend but no significant difference between the 10-29 years, 30-49 years, and 50-69 years groups (p> 0.05). When it comes to 70-89 years group, the ratios were decreased significantly compared with other three groups (p<0.01). Ratios derived from the improved method also had no significant difference between the first three groups (p> 0.05), but for the 80 years, the ratios were decreased significantly compared with 10-29 years group (p<0.05).  Conclusions: The parameters \|BD\| and TI proposed by Shifman  and channel were significantly influenced by age. The measurements of Seow and Lai (CB/R ratios) were less affected by age compared with the former. The  improved method(B/R ratios) was least affected by age, which would reduce error and bias in the measurement of taurodontism and get more objective results in older patients. |
| **Order of Authors:** | Yunmeng Da |
|  | Le Zhang |
|  | Zhihong Chai |
|  | Hongfang Du |
|  | LeLe Hao |
|  | Li Zhang |
|  | Zhiyin Zhang |
|  | Yongfan Shen |

*Powered by Editorial Manager® and ProduXion Manager® from Aries Systems Corporation*

| **Additional Information:** |  |
| --- | --- |
| **Question** | **Response** |
| **Financial Disclosure**  Enter a financial disclosure statement that describes the sources of funding for the work included in this submission. Review the [submission guidelines](http://journals.plos.org/plosone/s/submission-guidelines#loc-financial-disclosure-statement) for detailed requirements. View published research articles from [PLOS ONE](https://journals.plos.org/plosone/) for specific examples.  This statement is required for submission and **will appear in the published article** if the submission is accepted. Please make sure it is accurate. | Yes |
| **Funded studies**  Enter a statement with the following details:   - Initials of the authors who received each award - Grant numbers awarded to each author - The full name of each funder - URL of each funder website - Did the sponsors or funders play any role in the study design, data collection and analysis, decision to publish, or preparation of the manuscript? |  |
| Did you receive funding for this work? |  |
| Please add funding details.  as follow-up to "**Financial Disclosure**  Enter a financial disclosure statement that describes the sources of funding for the work included in this submission. Review the [submission guidelines](http://journals.plos.org/plosone/s/submission-guidelines#loc-financial-disclosure-statement) for detailed requirements. View published research articles from [PLOS ONE](https://journals.plos.org/plosone/) for specific examples.  This statement is required for submission and **will appear in the published article** if the submission is accepted. Please make sure it is accurate. | Xingtai Science and Technology Project, 2022zz081, Yun-meng Da |

*Powered by Editorial Manager® and ProduXion Manager® from Aries Systems Corporation*

| **Funded studies**  Enter a statement with the following details:   - Initials of the authors who received each award - Grant numbers awarded to each author - The full name of each funder - URL of each funder website - Did the sponsors or funders play any role in the study design, data collection and analysis, decision to publish, or preparation of the manuscript? |  |
| --- | --- |
| Did you receive funding for this work?" |  |
| Please select the country of your main research funder (please select carefully as in some cases this is used in fee calculation).  as follow-up to "**Financial Disclosure**  Enter a financial disclosure statement that describes the sources of funding for the work included in this submission. Review the [submission guidelines](http://journals.plos.org/plosone/s/submission-guidelines#loc-financial-disclosure-statement) for detailed requirements. View published research articles from [PLOS ONE](https://journals.plos.org/plosone/) for specific examples.  This statement is required for submission and **will appear in the published article** if the submission is accepted. Please make sure it is accurate. | CHINA - CN |
| **Funded studies**  Enter a statement with the following details:   - Initials of the authors who received each award - Grant numbers awarded to each author - The full name of each funder - URL of each funder website - Did the sponsors or funders play any role in the study design, data collection and analysis, decision to publish, or preparation of the manuscript? |  |
| Did you receive funding for this work?" |  |
| **Competing Interests** | The authors have declared that no competing interests exist. |

*Powered by Editorial Manager® and ProduXion Manager® from Aries Systems Corporation*

| Use the instructions below to enter a competing interest statement for this submission. On behalf of all authors, disclose any [competing interests](http://journals.plos.org/plosone/s/competing-interests) that could be perceived to bias this work—acknowledging all financial support and any other relevant financial or non- financial competing interests.  This statement is required for submission and **will appear in the published article** if the submission is accepted. Please make sure it is accurate and that any funding sources listed in your Funding Information later in the submission form are also declared in your Financial Disclosure statement.  View published research articles from  [PLOS ONE](http://journals.plos.org/plosone/) for specific examples. |  |
| --- | --- |
| **NO authors have competing interests**  Enter: The authors have declared that no competing interests exist.  **Authors with competing interests**  Enter competing interest details beginning with this statement:  I have read the journal's policy and the authors of this manuscript have the following competing interests: [insert competing interests here] |  |
| * typeset |  |
| **Ethics Statement**  Enter an ethics statement for this submission. This statement is required if the study involved:   - Human participants - Human specimens or tissue - Vertebrate animals or cephalopods - Vertebrate embryos or tissues | The study was approved by the medical ethics committee of Hebei Eye Hospital (No.: 2019KY002；No.: 2022KY14). The consent was not obtained , because only the CBCT images were collected, and the data were analyzed anonymously. |

*Powered by Editorial Manager® and ProduXion Manager® from Aries Systems Corporation*

| - Field research   Write "N/A" if the submission does not require an ethics statement.  General guidance is provided below. Consult the [submission guidelines](http://journals.plos.org/plosone/s/submission-guidelines#loc-guidelines-for-specific-study-types) for detailed instructions. **Make sure that all information entered here is included in the Methods section of the manuscript.** |  |
| --- | --- |

*Powered by Editorial Manager® and ProduXion Manager® from Aries Systems Corporation*

| **Format for specific study types**  **Human Subject Research (involving human participants and/or tissue)**   - Give the name of the institutional review board or ethics committee that approved the study - Include the approval number and/or a statement indicating approval of this research - Indicate the form of consent obtained (written/oral) or the reason that consent was not obtained (e.g. the data were analyzed anonymously)   **Animal Research (involving vertebrate animals, embryos or tissues)**   - Provide the name of the Institutional Animal   Care and Use Committee (IACUC) or other relevant ethics board that reviewed the study protocol, and indicate whether they approved this research or granted a formal waiver of ethical approval   - Include an approval number if one was obtained - If the study involved non-human primates, add additional details about animal welfare and steps taken to ameliorate suffering - If anesthesia, euthanasia, or any kind of animal sacrifice is part of the study, include briefly which substances and/or methods were applied   **Field Research**  Include the following details if this study involves the collection of plant, animal, or other materials from a natural setting:   - Field permit number - Name of the institution or relevant body that granted permission |  |
| --- | --- |
|  |  |
| **Data Availability**  Authors are required to make all data underlying the findings described fully available, without restriction, and from the time of publication. PLOS allows rare exceptions to address legal and ethical concerns. See the [PLOS Data Policy](http://journals.plos.org/plosone/s/data-availability) and [FAQ](http://journals.plos.org/plosone/s/data-availability#loc-faqs-for-data-policy) for detailed information. | Yes - all data are fully available without restriction |

*Powered by Editorial Manager® and ProduXion Manager® from Aries Systems Corporation*

| A Data Availability Statement describing where the data can be found is required at submission. Your answers to this question constitute the Data Availability Statement and **will be published in the article**, if accepted. |  |
| --- | --- |
| **Important:** Stating ‘data available on request from the author’ is not sufficient. If your data are only available upon request, select ‘No’ for the first question and explain your exceptional situation in the text box. |  |
| Do the authors confirm that all data underlying the findings described in their manuscript are fully available without restriction? |  |
| **Describe where the data may be found in full sentences. If you are copying our sample text, replace any instances of XXX with the appropriate details.**   - If the data are **held or will be held in a public repository**, include URLs, accession numbers or DOIs. If this information will only be available after acceptance, indicate this by ticking the box below. For example: All XXX files are available from the XXX database (accession number(s) XXX, XXX.). - If the data are all contained **within the**   **manuscript and/or Supporting Information files**, enter the following: All relevant data are within the manuscript and its Supporting Information files.   - If neither of these applies but you are able to provide **details of access elsewhere**, with or without limitations, please do so. For example:   Data cannot be shared publicly because of [XXX]. Data are available from the XXX Institutional Data Access / Ethics Committee (contact via XXX) for researchers who meet the criteria for access to confidential data.  The data underlying the results presented in the study are available from (include the name of the third party | All relevant data are within the manuscript and its Supporting Information files. |

*Powered by Editorial Manager® and ProduXion Manager® from Aries Systems Corporation*

| and contact information or URL).   - This text is appropriate if the data are owned by a third party and authors do not have permission to share the data.   * typeset |  |
| --- | --- |
| Additional data availability information: | Tick here if your circumstances are not covered by the questions above and you need the journal’s help to make your data available. |

*Powered by Editorial Manager® and ProduXion Manager® from Aries Systems Corporation*

<https://www.editorialmanager.com/pone/download.aspx?id=35162611&guid=5e0e787b-a711-476f-9149-e2aa381430c1&scheme=1>Manuscript	[Click here to access/download;Manuscript;Manuscript new.docx](https://www.editorialmanager.com/pone/download.aspx?id=35162611&guid=5e0e787b-a711-476f-9149-e2aa381430c1&scheme=1)

# 1 An improved diagnostic method for taurodontism and a

2 **comparative study on its effectiveness evaluation**
3 **Yunmeng Da^1*^, Le Zhang^2^, Zhihong Chai^1^, Hongfang Du^1^, Lele Hao^1^, Li Zhang^1^, Zhiyin Zhang^1^,**
4 **Yongfan Shen^1*^**
5 **Short Title:	An improved diagnostic method for taurodontism.**
6 1Department of prothodontics, Hebei Eye Hosipital; Hebei Provincial Key Laboratory of
7 Ophthalmology; Hebei Clinical Medical Research Center of Eye Diseases, Xingtai, China
8 2Department of Oral and Maxillofacial Surgery, Hebei Eye Hosipital; Hebei Provincial Key Laboratory
9 of Ophthalmology; Hebei Clinical Medical Research Center of Eye Diseases, Xingtai, China
10 * corresponding author
11 **Conflict of interest** No conflict of interest.
12 **Ethical statements** The study was approved by the medical ethics committee of Hebei Eye Hospital
13 (No.: 2022KY14). The consent was not obtained , because only the CBCT images were collected, and
14 the data were analyzed anonymously.
15 **Adress correspondence to:**
16 1.Yunmeng Da
17 Department of prothodontics
18 Hebei Eye Hosipital; Hebei Provincial Key Laboratory of Ophthalmology; Hebei Clinical Medical
19 Research Center of Eye Diseases.
20 Quanbei East Street,No.:399,Xiangdu District, Xingtai, China
21 Tel:+86 15631900791
22 mail: [dayunmeng@126.com](mailto:dayunmeng@126.com)
23 2.Le Zhang
24 Department of Oral and Maxillofacial Surgery, Hebei Eye Hosipital；Hebei Provincial Key Laboratory
25 of Ophthalmology；Hebei Clinical Medical Research Center of Eye Diseases
26 Quanbei East Street,No.:399,Xiangdu District, Xingtai, China
27 Tel:+86 15631900927
28 mail: [350121459@qq.com](mailto:dayunmeng@126.com)
29 Zhihong Chai
30 Department of prothodontics
31 Hebei Eye Hosipital; Hebei Provincial Key Laboratory of Ophthalmology; Hebei Clinical Medical
32 Research Center of Eye Diseases
33 Quanbei East Street,No.:399,Xiangdu District, Xingtai, China
34 Tel:+86 15631901629
35 E-mail: [38176970@qq.com](mailto:dayunmeng@126.com)

36

37 Hongfang Du
38 Department of prothodontics
39 Hebei Eye Hosipital; Hebei Provincial Key Laboratory of Ophthalmology; Hebei Clinical Medical
40 Research Center of Eye Diseases
41 Quanbei East Street,No.:399,Xiangdu District, Xingtai, China
42 Tel:+86 15631901032
43 E-mail: [duhongfangxfk@126.com](mailto:dayunmeng@126.com)
44 Lele Hao
45 Department of prothodontics
46 Hebei Eye Hosipital; Hebei Provincial Key Laboratory of Ophthalmology; Hebei Clinical Medical
47 Research Center of Eye Diseases
48 Quanbei East Street,No.:399,Xiangdu District, Xingtai, China
49 Tel:+86 15631901399
50 mail: [965652177@qq.com](mailto:dayunmeng@126.com)
51 Li Zhang
52 Department of prothodontics
53 Hebei Eye Hosipital; Hebei Provincial Key Laboratory of Ophthalmology; Hebei Clinical Medical
54 Research Center of Eye Diseases
55 Quanbei East Street,No.:399,Xiangdu District, Xingtai, China
56 Tel:+86 15631901768
57 mail: [416720157@qq.com](mailto:dayunmeng@126.com)
58 Zhiyin Zhang
59 Department of prothodontics
60 Hebei Eye Hosipital；Hebei Provincial Key Laboratory of Ophthalmology；Hebei Clinical Medical
61 Research Center of Eye Diseases
62 Quanbei East Street,No.:399,Xiangdu District, Xingtai, China
63 Tel:+86 15631901678
64 E-mail: [15631900791@163.com](mailto:dayunmeng@126.com)
65 8. Yongfan Shen
66 Department of prothodontics
67 Hebei Eye Hosipital; Hebei Provincial Key Laboratory of Ophthalmology; Hebei Clinical Medical
68 Research Center of Eye Diseases
69 Quanbei East Street,No.:399,Xiangdu District, Xingtai, China
70 Tel:+86 15631901179
71 E-mail: [syf200506@163.com](mailto:dayunmeng@126.com)

72

73 **Keywords:** Taurodontism; Cone-beam computed tomography; Dental malformations; Taurodontism
74 index; Crown-body/root ratios.

75

76

# 77 Abstract

78 **Objective:** Since the two commonly used diagnostic methods of
79 taurodontism were susceptible to aging changes, mastication wear and
80 other factors, this study proposed an improved diagnostic method of
81 taurodontism, and compared with the previous two methods, as a
82 supplementary for the taurodontism diagnosis.
83 **Methods:** The patients included were age 10–89 years and admitted to
84 the Department of Stomatology of Hebei Eye Hospital from June 1, 2022
85 to May 31, 2023. 80 CBCT images were selected and divided into 4
86 groups: 10-29 years, 30-49 years, 50-69 years, and 70-89 years, with 20
87 images in each group. Select the right mandibular first molars as the
88 measurement object. Firstly,	|BD| and taurodontism index (TI) related
89 parameters |AB| and |AC| were measured using Shifman and Chanannel's
90 method and the length of crow-body(CB) and root (R) was measured by
91 Seow and Lai's method. Then the improved method took the length of
92 CEJ to root bifurcation point（Body，B） and the root length（Root，R）
93 as the measurement objects. Finally, TI ,CB/R ratios and B/R ratios was
94 calculated according to the formulas given. One-way ANOVA analysis
95 was mainly used to compare the differences in the values, indexes or
96 ratios of taurodontism among different age groups (P<0.05).
97 **Results:** Among groups, with the increase of age, the values of |BD| and
98 TI decreased significantly (p<0.01). For CB/R ratios, there was a
99 downward trend but no significant difference between the 10-29 years,
100 30-49 years, and 50-69 years groups (p> 0.05). When it comes to 70-89
101 years group, the ratios were decreased significantly compared with other
102 three groups (p<0.01). Ratios derived from the improved method also had
103 no significant difference between the first three groups (p> 0.05), but for
104 the 80 years, the ratios were decreased significantly compared with 10-29
105 years group (p<0.05).
106 **Conclusions:** The parameters |BD| and TI proposed by Shifman and
107 channel were significantly influenced by age. The measurements of Seow
108 and Lai (CB/R ratios) were less affected by age compared with the former.
109 The improved method(B/R ratios) was least affected by age, which would
110 reduce error and bias in the measurement of taurodontism and get more
111 objective results in older patients.
112 **Keywords:** Taurodontism; Diagnostic method; CEJ; Root bifurcation;
113 Taurodontism index; Crown-body/root ratios.

# 114 Introduction

115 In 1913, Keith proposed the concept of "taurodont" [1], then it was

116classified into hypotaurodontism, mesotaurodontism, hypertaurodontism by

117Shaw et al. [2]. Time to 1978, taurodontism was diagnosed by measuring the

118|BD| values and taurodontism index (TI) on oral radio graph[3]. About a

119decade later, Seow and Lai used the CB/R ratio as an indicator for the

120evaluation of taurodontism[4]. So far, the two methods were commonly used

121in most studies [5,6,7,8].

122	Reviewing the previous studies, we noticed that the prevalence of

123taurodontism varied greatly among different races and countries. The lo

124west incidence of taurodontism can be as low as 0.1% in western Sau

125di Arabia population [9,10], but the highest was as high as 52.4% in

126Chinese[6].	Previous research by our group has found that the imprope

127r selection of diagnostic methods for taurodontism was the real reason

128for the high and low incidence of taurodontism between different count

129ries or populations. The two commonly used measurement methods of

130taurodontism were affected by aging changes, mastication wear and oth

131er factors, leading to the bias of measurement results. In this study, in

132order to provide a more accurate measurement method and standard for

133 the diagnosis of taurodontism which was less affected by age, we im

134proved the CB/R ratio method and used CEJ point to replace the lowe

135st point of the dental crown, and compared it with the previous two to

136 evaluate its effectiveness.

## 137 1.Methods

138 **1.1 Subjects**
139 The study was approved by the medical ethics committee of Hebei Eye
140 Hospital (No.: 2019KY002；No.: 2022KY14). The consent was not
141 obtained , because only the CBCT images were collected, and the data
142 were analyzed anonymously. Only authors can access to information that
143 could identify individual participants during or after data collection. The
144 patients included were age 10–89 years and admitted to the Department
145 of Stomatology of Hebei Eye Hospital from June 1, 2022 to May 31,
146 2023. Starting on June 1, 2023 and took about two weeks, data were
147 accessed for research purposes. The inclusion criteria: mandibular first
148 molars, with fully developed roots, and no obvious caries or tooth defects
149 affected the measurement. We collected 80 CBCT images randomly aged
150 between 10 and 89 years and divided into 4 groups: 10-29 years, 30-49
151 years, 50-69 years, 70-89 years, with 20 images in each group. Select the
152 right mandibular first molar as the measurement object.

## 153 1.2 Diagnosis criteria

154 Firstly, 80 tooth were measured using the method proposed by Shifman

155& channel to obtain |BD| and TI values. Method: |BD| was the vertical

156distance from the CEJ to the highest point of the pulp chamber

157floor. TI=|AB|/|AC|×100, where |AB| was the vertical distance from the

158lowest point of the plasma chamber ceiling to the highest point of the plasma

159chamber floor, and |AC| was the vertical distance from the lowest point of

160ceiling to the longest root tip (Figure 1).

161

162	Figure 1.The measurements of Shifman & channel: |BD| is the vertical distance from the CEJ

163	to the highest point at the bottom of the pulp chamber. |AB| is the vertical distance between the

164	lowest point at the top of the pulp chamber and the highest point at the bottom of the pulp

165	chamber; |AC| is the perpendicular distance between the lowest point at the top of the pulp

166	chamber and the apex of the longest root.

167

168	After that, 80 tooth were measured again using the measurement

169methods of Seow and Lai. CB refers to the vertical distance from the lowest

170point of the occlusion surface to the root bifurcation, and R refers to the

171vertical distance from the root bifurcation to the longest root tip. CB and R

172values related to the CB/R ratio were obtained, we divided CB and R get

173CB/R ratios. (Figure 2) [4].

174	Finally，80 tooth were measured using the improved method. B refers to

175the vertical distance from CEJ to the root bifurcation, and R refers to the

176vertical distance from the root bifurcation to the longest root tip. Just like the

177CB/R ratio, B and R values related to B/R ratio, and divided B and R get B/R

178ratios(Figure 2) .

179

180 Figure 2．The measurements of CB/R ratio and B/R ratio:Crown（C）is the vertical distance
181 from the lowest point of occlusion surface to the CEJ. Body（B）is the vertical distance from the
182 CEJ to the Root bifurcation point. and Root（R）is the vertical distance from the root bifurcation
183 point to the longest root tip.

184

## 185 1.3 Analysis and statistics

186 Firstly, the values of |BD|, |AB|, |AC|, CB, B and R measured by CBCT

187using Image Pro plus 6.0 software. The index were obtained according to the

188corresponding calculation formula. Spss16.0 software analysis data, P<0.05

189means significant. The homogeneity test of variance showed that the levels of

190variance in the TI was heterogeneous using Levene test(P<0.05), the

191Kruskal-Wallis H test and Mann-Whitney U tests were used to analyze these

192data. Other data conformed to normal distribution(P>0.05), were performed

193by one-way ANOVA and LSD test.

## 194 2. Results

195 With the increase of age, the values of |BD| decreased significantly

196(p<0.01) (Figure 3). The TI decreased significantly with aging

197too(p< 0.01)(Figure 4). In the first three age groups, CB/R ratios tended to

198decrease but were no significant difference(p> 0.05), until the 70-89 age

199group showed a significant decline compared to the other groups(p< 0.01)

200(Figure 5). B/R ratio also showed a downward trend, but only the 70-89 age

201group showed a significant decline compared to the 10-29

202group(p< 0.05)(Figure 6).The data can be viewed in Table 1.

203

|  | methods |  | | | |
| --- | --- | --- | --- | --- | --- |
|  |  | 10-29 years | 30-49 years | 50-69 years | 70-89 years |
|  | \|BD\| | 1.54±0.43 | 0.98±0.38 | 0.81±0.31 | 0.45±0.23 |
|  | TI | 11.93±2.69 | 7.29±3.56 | 4.27±2.37 | 3.10±1.24 |
|  | CB/R | 0.92±0.11 | 0.88±0.09 | 0.8685±0.09 | 0.75±0.08 |
|  | B/R | 0.47±0.07 | 0.44±0.07 | 0.43±0.09 | 0.41±0.07 |
| 204 |  |  |  |  |  |
| 205 |  |  |  |  |  |

206	Table1：The |BD|, TI ,CB/R and B/R values of the mandibular first molar were (X ± s).

207

208 Figure 3. The variation trend of |BD| value with age: with the increase of age, the value of |BD|
209 decreased significantly (p<0.01) . * stand for p<0.01 vs other groups. ● stand for p<0.01 vs other
210 groups except 50-69 years group(p>0.05). ■ stand for p<0.01 vs other groups except 30-49 years
211 group(p>0.05). ★ stand for p<0.01 vs other groups.

212

213 Figure4. Trend of TI values with age: The TI values decreased significantly with the increase of
214 age, * stand for p<0.01 vs other groups. ■ stand for p<0.01 vs other groups except 70-89 years
215 groups(p>0.05). ★ stand for p<0.01 vs other groups except 50-69 years groups(p>0.05).

216

217 Figure5. Trend of CB/R ratios with years: There was no significant difference of CB/R value
218 between the first three groups(p<0.05). 70-89 group was decreased vs the other groups
219 significantly. * means p<0.01 vs the other groups.

220

221 Figure 6. Trend of B/R ratios with years: There was no significant difference of CB/R value
222 between the first three groups(p<0.05). 70-89 group was decreased vs 10-29 groups significantly.
223 * means p<0.01 vs the other groups.

## 224 3. Classification of the improved method.

225 Since the modified method was an improvement on the basis of CB/R

226ratio,	we	still	used	the	grade	conversion	method	of	CB/R	ratio,

227hypotaurodontism(CB/R≤1.10),	mesotaurodontism(1.30<CB/R≤2.0),

228hypertaurodontism (2.0<CB/R) [4]. It was estimated that the CB/R ratio is

229about	twice	that	of	B/R,	and	based	on	this,	we	conclude	that

230hypotaurodontism	(B/R≤0.55),	mesotaurodontism	(0.55<B/R≤0.65),

231hypertaurodontism (1.0 < B/R) .

232	**Disscussion**

233	Through the analysis of the research results, it was found that the values

234of |BD| and TI in Shifman & channel's method were significantly affected by

235age and showed a downward trend sharply, which would undoubtedly lead to

236bias in the measurement of taurodontism, and false negative results would

237occur in the measurement of older patients. The result was easy to explain,

238with the increase of age, the top point of the pulp chamber was down growth,

239and the bottom point was up growth, resulting in a reduction in the overall

240height of the pulp cavity. |BD| was the distance from the CEJ to the pulp

241chamber floor. Although the position of the CEJ does not change, the increase

242of the pulp chamber floor lead to the decrease of |BD|. The TI related to the

243height of the top and bottom of the pulp chamber, so it had been significantly

244reduced with aging. Many statistics on the incidence of taurodontism had

245adopted the Shifman & channel method, some of which selected adolescents

246as research objects and get relatively accurate results, while others targeted at

247different age groups, including middle-aged and elderly people. It was

248obviously inappropriate to adopt this method, which will inevitably lead to an

249underestimation	of	the	incidence. Obviously,	taurodontism	should	not

250disappear with age.

251	The measurements of Seow and Lai (CB/R ratio) were less affected by

252age compared with Shifman & channel's method. This was mainly because

253the lowest point of the occlusion surface was selected in this method.

254Although the occlusion surface would also decrease with chewing wear, the

255process would take decades to the lowest point. After 70 years of age, the

256CB/R ratio decreased significantly because the enamel of the occlusion

257surface was worn out, the softer dentin was exposed, and the wear rate

258accelerated. However, it was not ruled out that some patients with acidosis or

259gastroesophageal reflux disease which would have severe wear in advance

260due to acid erosion of dental minerals, resulting in low measurement values

261also.

262	The improved method proposed by us uses CEJ points as markers. On

263the one hand, CEJ point was not affected by age-related changes after

264formed; on the other hand, CEJ points are generally located below the gums,

265and factors such as wear or acidosis rarely affect them, so the diagnosis of

266taurodontism based on it was more accurate and objective. We observed that

267compared with the first two methods, the improved method was least

268affected by age. Only the age group of 70-89 years and 10-29 years old

269showed a significant decline. We believe that this might be related to the

270different dietary habits and food types of the two generations. The growth

271and development period of the elderly was the transition of the old and new

272society in China, when the food was mainly coarse grains and the

273mastication force was strong. The stimulation of tooth root development was

274longer, the root bifurcation was larger, lead to the reduce of B/R ratios. The

275growth and development period of 10-29 years old was in the modern era

276with abundant fine soft food, the mastication force was weaker than 70-89

277years	group,	shorter	tooth	root	development,	and	the	ratio	was

278correspondingly enhanced.

279	Taurodontism posed challenges to the treatment of various dental

280disciplines, including endodontic diseases, periodontal diseases, orthodontics

281and prosthetics [8], so it was extremely important to obtain accurate

282incidence rates. To sum up, the selection of diagnostic methods for

283taurodontism should be based on different age groups to choose different

284detection methods. The Shifman & channel method can be used to measure

285juveniles with fully developed root tips. For middle-aged patients, Seow and

286Lai’s method was chosen as far as possible. The improved method was more

287practical for elderly patients, with severe mastication wear or obvious

288depression of the occlusion surface. The study proposed an improved

289diagnostic method for taurodontism and a comparative study on its

290effectiveness evaluation and provided a reference for clinicians to accurately

291diagnose and treat taurodontism in clinical practice.

292 **Funding**

293Xingtai Science and Technology Project, NO.: 2022zz081, Yun-meng Da

## 294Declarations

295Conflict of interest No conflict of interest.

296

297 **References**
298 1. Keith A. Problems relating to the Teeth of the Earlier Forms of Prehistoric Man. Proc R Soc
299 Med. 1913;6(Odontol Sect):103-24. PMID: 19977113; PMCID: PMC2005996.
300 2. Shaw JC. Taurodont Teeth in South African Races. J Anat. 1928 Jul;62(Pt 4):476-498.1. PMID:
301 17104204; PMCID: PMC1249989.
302 3. Shifman A, Chanannel I. Prevalence of taurodontism found in radiographic dental examination
303 of 1,200 young adult Israeli patients. Community Dent Oral Epidemiol. 1978 Jul;6(4):200-3. doi:
304 10.1111/j.1600-0528.1978.tb01150.x. PMID: 278704.
305 4. Seow WK, Lai PY. Association of taurodontism with hypodontia: a controlled study. Pediatr
306 Dent. 1989 Sep;11(3):214-9. PMID: 2638007.
307 5. MacDonald-Jankowski DS, Li TT. Taurodontism in a young adult Chinese population.
308 Dentomaxillofac Radiol. 1993 Aug;22(3):140-4. doi: 10.1259/dmfr.22.3.8299833. PMID:
309 8299833.
310 6. Da Y , Wang C , Zhang L ,et al. High prevalence of taurodontism in North China and its
311 relevant	factors:	a	retrospective	cohort	study[J].	Oral	Radiology,
312 2022:1-9.DOI:10.1007/s11282-022-00630-1.
313 7. Li Y, Qian F, Wang D, Wang Y, Wang W, Tian Y. Prevalence of taurodontism in individuals in
314 Northwest China determined by cone-beam computed tomography images. Heliyon. 2023 Apr
315 15;9(4):e15531. doi: 10.1016/j.heliyon.2023.e15531. PMID: 37128323; PMCID: PMC10148092.
316 8. Pach J, Regulski PA, Tomczyk J, Strużycka I. Clinical implications of a diagnosis of
317 taurodontism: A literature review. Adv Clin Exp Med. 2022 Dec;31(12):1385-1389. doi:
318 10.17219/acem/152120. PMID: 36000881.
319 9. Afify AR, Zawawi KH. The prevalence of dental anomalies in the Western region of saudi
320 arabia. ISRN Dent. 2012;2012:837270. doi: 10.5402/2012/837270. Epub 2012 Jun 19. PMID:
321 22778974; PMCID: PMC3388344.
322 10. Colak H, Tan E, Bayraktar Y, Hamidi MM, Colak T. Taurodontism in a central anatolian
323 population. Dent Res J (Isfahan). 2013 Mar;10(2):260-3. doi: 10.4103/1735-3327.113367. PMID:
324 23946746; PMCID: PMC3731970.

Figure1	[Click here to access/download;Figure;Figure 1.tif](https://www.editorialmanager.com/pone/download.aspx?id=34908003&guid=22ae172b-e519-4d3c-8e7c-343b6a8943e7&scheme=1)[<https://www.editorialmanager.com/pone/download.aspx?id=34908003&guid=22ae172b-e519-4d3c-8e7c-343b6a8943e7&scheme=1>](https://www.editorialmanager.com/pone/download.aspx?id=34908003&guid=22ae172b-e519-4d3c-8e7c-343b6a8943e7&scheme=1)

Figure2	[Click here to access/download;Figure;Figure 2.tif](https://www.editorialmanager.com/pone/download.aspx?id=34908004&guid=0da26170-4d3d-4702-b7ea-dfbe94a59a24&scheme=1)[<https://www.editorialmanager.com/pone/download.aspx?id=34908004&guid=0da26170-4d3d-4702-b7ea-dfbe94a59a24&scheme=1>](https://www.editorialmanager.com/pone/download.aspx?id=34908004&guid=0da26170-4d3d-4702-b7ea-dfbe94a59a24&scheme=1)

Figure3	[Click here to access/download;Figure;Figure 3.tif](https://www.editorialmanager.com/pone/download.aspx?id=34908005&guid=02368a68-1148-4e5d-a471-44b517de6bac&scheme=1)[<https://www.editorialmanager.com/pone/download.aspx?id=34908005&guid=02368a68-1148-4e5d-a471-44b517de6bac&scheme=1>](https://www.editorialmanager.com/pone/download.aspx?id=34908005&guid=02368a68-1148-4e5d-a471-44b517de6bac&scheme=1)

Figure4	[Click here to access/download;Figure;Figure 4.tif](https://www.editorialmanager.com/pone/download.aspx?id=34908006&guid=60881d6a-6f75-4e55-b1f1-6d1acf8c40da&scheme=1)[<https://www.editorialmanager.com/pone/download.aspx?id=34908006&guid=60881d6a-6f75-4e55-b1f1-6d1acf8c40da&scheme=1>](https://www.editorialmanager.com/pone/download.aspx?id=34908006&guid=60881d6a-6f75-4e55-b1f1-6d1acf8c40da&scheme=1)

Figure5	[Click here to access/download;Figure;Figure 5.tif](https://www.editorialmanager.com/pone/download.aspx?id=34908007&guid=701190b3-d0c6-4739-8629-98ce34c20a0f&scheme=1)[<https://www.editorialmanager.com/pone/download.aspx?id=34908007&guid=701190b3-d0c6-4739-8629-98ce34c20a0f&scheme=1>](https://www.editorialmanager.com/pone/download.aspx?id=34908007&guid=701190b3-d0c6-4739-8629-98ce34c20a0f&scheme=1)

Figure6	[Click here to access/download;Figure;Figure 6.tif](https://www.editorialmanager.com/pone/download.aspx?id=34908009&guid=8e765b10-4c78-4922-8586-42bb6b9cb37a&scheme=1)[<https://www.editorialmanager.com/pone/download.aspx?id=34908009&guid=8e765b10-4c78-4922-8586-42bb6b9cb37a&scheme=1>](https://www.editorialmanager.com/pone/download.aspx?id=34908009&guid=8e765b10-4c78-4922-8586-42bb6b9cb37a&scheme=1)

Supporting Information

[Click here to access/download](https://www.editorialmanager.com/pone/download.aspx?id=34908011&guid=de1ccb49-8fab-4e3e-9af7-eb3e79f9c565&scheme=1)

[**Supporting Information** Ethics Approval 1.pdf](https://www.editorialmanager.com/pone/download.aspx?id=34908011&guid=de1ccb49-8fab-4e3e-9af7-eb3e79f9c565&scheme=1)

Supporting Information

[Click here to access/download](https://www.editorialmanager.com/pone/download.aspx?id=34908013&guid=045b7f37-d288-4d75-a4d0-9798b80020d0&scheme=1)

[**Supporting Information** Ethics Approval 1 translation.docx](https://www.editorialmanager.com/pone/download.aspx?id=34908013&guid=045b7f37-d288-4d75-a4d0-9798b80020d0&scheme=1)

Supporting Information

[Click here to access/download](https://www.editorialmanager.com/pone/download.aspx?id=34908014&guid=880ad3ee-6187-4d64-9d57-2880aee368b9&scheme=1)

[**Supporting Information** Ethics Approval 2.jpg](https://www.editorialmanager.com/pone/download.aspx?id=34908014&guid=880ad3ee-6187-4d64-9d57-2880aee368b9&scheme=1)

Supporting Information

[Click here to access/download](https://www.editorialmanager.com/pone/download.aspx?id=34908015&guid=5da517d9-59c8-4073-9409-fa05b9f7f017&scheme=1)

[**Supporting Information** Ethics Approval 2 translation.docx](https://www.editorialmanager.com/pone/download.aspx?id=34908015&guid=5da517d9-59c8-4073-9409-fa05b9f7f017&scheme=1)
