# Supplementary material for: An improved diagnostic method for taurodontism and a comparative study on its effectiveness evaluation
Source: PLoS One. 2024 May 7;19(5):e0302810. doi: 10.1371/journal.pone.0302810 (PMC11075821; doi:10.1371/journal.pone.0302810)
Supplement: Attachment — Submitted filename: Review letter.docx [file pone.0302810.s003.docx]

**Review letter**

**Reviewer Name: Prof. Dr. Dena Nadhim Mohammad**

**Ph.D -Oral Pathology**

**Head of Oral Diagnosis Department**

**College Of Dentistry -University of Sulaimani, Kurdistan Region, Iraq**

**Minor corrections should be made.**

The manuscript needs minor grammar checking with proper punctuation.

The authors should make the required corrections.

**1-Abstract**

The abstract is well-organized and clear.

The authors should make the required corrections that are highlighted.

**2- Introduction**

1. The authors should make the required corrections that are highlighted.
2. Give a definition of taurodontism and add refs to the introduction.

**3-Material and method**

1. The author should make the required corrections that are highlighted.
2. The author should mention the country of study to compare the study's findings with those of other countries in the discussion.

**4- Results**

1. The author should make the required corrections that are highlighted.
2. The author should change Fig 6, as a slight increase in B/R ratio was seen in the 50-69 age group compared to 30 -49, as shown in Fig 6.
3. In a classification of the improved method, correct symbols.
4. In a classification of the improved method, the authors should mention their results of CB/R and B/R according to this grade.

**5- Discussion**

1. The author should make the required corrections that are highlighted.
2. The author should put refs for other studies that used Shifman & channel method to determine taurodontism.
3. The authors should discuss their research findings regarding the (grade) of taurodontism of improved classification B/R , compare results to other countries, explain the cause of the difference, and add refs.
4. The author should add other references to the discussion (The Shifman & channel method can be used to measure juveniles with fully developed root tips. For middle-aged patients, Seow and Lai’s method was chosen as far as possible)

**6-	References**

1. The author should make the required corrections that are highlighted.
2. Add other references, as the number of refs is few.
