# Supplementary material for: An improved diagnostic method for taurodontism and a comparative study on its effectiveness evaluation
Source: PLoS One. 2024 May 7;19(5):e0302810. doi: 10.1371/journal.pone.0302810 (PMC11075821; doi:10.1371/journal.pone.0302810)
Supplement: Attachment — Submitted filename: Responds to Reviewers.docx [file pone.0302810.s004.docx]

Journal Requirements:

When submitting your revision, we need you to address these additional requirements.

1. Please ensure that your manuscript meets PLOS ONE's style requirements, including those for file naming. The PLOS ONE style templates can be found at

https://journals.plos.org/plosone/s/file?id=wjVg/PLOSOne_formatting_sample_main_body.pdf and 
<https://journals.plos.org/plosone/s/file?id=ba62/PLOSOne_formatting_sample_title_authors_affiliations.pdf>

**Answer：**It has been modified according to your suggestion.

2. We suggest you thoroughly copyedit your manuscript for language usage, spelling, and grammar. If you do not know anyone who can help you do this, you may wish to consider employing a professional scientific editing service.

Whilst you may use any professional scientific editing service of your choice, PLOS has partnered with both American Journal Experts (AJE) and Editage to provide discounted services to PLOS authors. Both organizations have experience helping authors meet PLOS guidelines and can provide language editing, translation, manuscript formatting, and figure formatting to ensure your manuscript meets our submission guidelines. To take advantage of our partnership with AJE, visit the AJE website (http://learn.aje.com/plos/) for a 15% discount off AJE services. To take advantage of our partnership with Editage, visit the Editage website (www.editage.com) and enter referral code PLOSEDIT for a 15% discount off Editage services.  If the PLOS editorial team finds any language issues in text that either AJE or Editage has edited, the service provider will re-edit the text for free.

Upon resubmission, please provide the following:

- The name of the colleague or the details of the professional service that edited your manuscript.
- A copy of your manuscript showing your changes by either highlighting them or using track changes (uploaded as a *supporting information* file).

A clean copy of the edited manuscript (uploaded as the new *manuscript* file).

**Answer**：Articles have been copyedited using Editage, and the language usage, spelling, and grammar have been optimized. The copyedit proofs have been uploaded to other materials. Traces are uploaded as a *supporting information* file. A clean copy of the edited manuscript have been uploaded as the new *manuscript* file.

3. Please be informed that funding information should not appear in the Acknowledgments section or other areas of your manuscript. We will only publish funding information present in the Funding Statement section of the online submission form. Please remove any funding-related text from the manuscript.

**Answer：**The funding information had been removed from the manuscript.

4. Thank you for stating the following financial disclosure: 
"Xingtai Science and Technology Project, 2022zz081, Yun-meng Da"
 
Please state what role the funders took in the study.  If the funders had no role, please state: "The funders had no role in study design, data collection and analysis, decision to publish, or preparation of the manuscript." If this statement is not correct you must amend it as needed.

Please include this amended Role of Funder statement in your cover letter; we will change the online submission form on your behalf.

**Answer：**The relevant statements have been made in the cover letter.

4. Your ethics statement should only appear in the Methods section of your manuscript. If your ethics statement is written in any section besides the Methods, please delete it from any other section.

**Answer：**We have checked the ethics statement in the article and only made it in the Methods section.

5. Please include captions for your Supporting Information files at the end of your manuscript, and update any in-text citations to match accordingly. Please see our Supporting Information guidelines for more information: http://journals.plos.org/plosone/s/supporting-information.

**Answer：**We have include captions for your Supporting Information files at the end of your manuscript.

6. Please review your reference list to ensure that it is complete and correct. If you have cited papers that have been retracted, please include the rationale for doing so in the manuscript text, or remove these references and replace them with relevant current references. Any changes to the reference list should be mentioned in the rebuttal letter that accompanies your revised manuscript. If you need to cite a retracted article, indicate the article’s retracted status in the References list and also include a citation and full reference for the retraction notice.

**Answer：** We have reviewed the reference carefully to ensure that it is complete and correct. We must emphasize that noretracted articles have been cited .
Changes to the reference were mentioned in the rebuttal letter that accompanies your revised manuscript.
**Additional Editor Comments:**

Greetings,
The author(s) should follow instruction suggested by peer reviewer.

**Answer：** Thank you for the advice, we have followed instruction suggested by peer reviewe, and carefully modified the errors in the article.

[Note: HTML markup is below. Please do not edit.]

Reviewers' comments:

Reviewer's Responses to Questions

**Comments to the Author**

1. Is the manuscript technically sound, and do the data support the conclusions?

The manuscript must describe a technically sound piece of scientific research with data that supports the conclusions. Experiments must have been conducted rigorously, with appropriate controls, replication, and sample sizes. The conclusions must be drawn appropriately based on the data presented.

Reviewer #1: Yes

2. Has the statistical analysis been performed appropriately and rigorously?

Reviewer #1: Yes

3. Have the authors made all data underlying the findings in their manuscript fully available?

The [PLOS Data policy](http://www.plosone.org/static/policies.action#sharing) requires authors to make all data underlying the findings described in their manuscript fully available without restriction, with rare exception (please refer to the Data Availability Statement in the manuscript PDF file). The data should be provided as part of the manuscript or its supporting information, or deposited to a public repository. For example, in addition to summary statistics, the data points behind means, medians and variance measures should be available. If there are restrictions on publicly sharing data—e.g. participant privacy or use of data from a third party—those must be specified.

Reviewer #1: Yes

4. Is the manuscript presented in an intelligible fashion and written in standard English?

PLOS ONE does not copyedit accepted manuscripts, so the language in submitted articles must be clear, correct, and unambiguous. Any typographical or grammatical errors should be corrected at revision, so please note any specific errors here.

Reviewer #1: Yes

5. Review Comments to the Author

Please use the space provided to explain your answers to the questions above. You may also include additional comments for the author, including concerns about dual publication, research ethics, or publication ethics. (Please upload your review as an attachment if it exceeds 20,000 characters)

Reviewer #1: Minor corrections should be made.
The manuscript needs minor grammar checking with proper punctuation.
The authors should make the required corrections.
**Answer：**The grammar and punctuation of the article have been revised. Thank you for your valuable comments.

1-Abstract
  The abstract is well-organized and clear.
  The authors should make the required corrections that are highlighted.
  **Answer:** It has been modified as required. Thank you for your valuable comments.
2-2- Introduction
  1. The authors should make the required corrections that are highlighted.
  2. Give a definition of taurodontism and add refs to the introduction.
  **Answer:** It has been modified as required, and the definition of taurodontism and references have been added in Page3, Line59-61 [1]. Thank you for your valuable suggestions.
  3-Material and method
  1. The author should make the required corrections that are highlighted.
  2. The author should mention the country of study to compare the study's findings with those of other countries in the discussion.
  **Answer:**It has been revised as requested, and the countries participating in the study have been added in P4, Line87. Thank you for your valuable comments.
  4- Results
  1. The author should make the required corrections that are highlighted.
  2. The author should change Fig 6, as a slight increase in B/R ratio was seen in the 50-69 age group compared to 30 -49, as shown in Fig 6.
  3. In a classification of the improved method, correct symbols.
  4. In a classification of the improved method, the authors should mention their results of CB/R and B/R according to this grade.
  **Answer:**The Highlight section has been modified. We must apologize for the error in Fig6, because of the rounding problem of the decimal point, we increased the decimal point by one place in Table1, and the age group of 50-69 compared to 30-49 did increase slightly. Thank you for your valuable advice. In P8, L179-184 have been correct symbols and stated the relationship between CB/R and B/R.

  5- Discussion
  1. The author should make the required corrections that are highlighted.
  2. The author should put refs for other studies that used Shifman & channel method to determine taurodontism.
  3. The authors should discuss their research findings regarding the (grade) of taurodontism of improved classification B/R , compare results to other countries, explain the cause of the difference, and add refs.
  4. The author should add other references to the discussion (The Shifman & channel method can be used to measure juveniles with fully developed root tips. For middle-aged patients, Seow and Lai’s method was chosen as far as possible)
  **Answer:** The highlight section has been revised. Thanks for your valuable comments. Regarding question 2, the literature used Shifman & channel method to determine taurodontism, adding refs[13] and [14]. As for question 3, because it is a newly proposed improved scheme, there is no existing research for comparison. The latest research progress of taurodontism is added in P9-10, and references are added. Question 4, (The Shifman & channel method can be used to measure juveniles with fully developed root tips. For middle-aged patients, Seow and Lai's method was chosen as far as possible) References were added.


  6- References
  1. The author should make the required corrections that are highlighted.
  2. Add other references, as the number of refs is few.

**Answer:** The Highlight section has been modified. We reviewed the latest research progress and added references according to the content of the article.

6. PLOS authors have the option to publish the peer review history of their article ([what does this mean?](https://journals.plos.org/plosone/s/editorial-and-peer-review-process#loc-peer-review-history)). If published, this will include your full peer review and any attached files.
**Answer:** No.

If you choose “no”, your identity will remain anonymous but your review may still be made public.


**Do you want your identity to be public for this peer review?** For information about this choice, including consent withdrawal, please see our [Privacy Policy](https://www.plos.org/privacy-policy).

Reviewer #1: No
